# *Bartonella henselae* in Porpoise Blood

**DOI:** 10.3201/eid1112.050969

**Published:** 2005-12

**Authors:** Ricardo G. Maggi, Craig A. Harms, Aleta A. Hohn, D. Ann Pabst, William A. McLellan, Wendy J. Walton, David S. Rotstein, Edward B. Breitschwerdt

**Affiliations:** * North Carolina State University College of Veterinary Medicine, Raleigh, North Carolina, USA; † Center for Marine Sciences and Technology, Morehead City, North Carolina, USA; ‡ National Marine Fisheries Service, Beaufort, North Carolina, USA; § University of North Carolina Wilmington, Wilmington, North Carolina, USA; ¶ Virginia Aquarium and Marine Science Center, Virginia Beach, Virginia, USA; # University of Tennessee College of Veterinary Medicine, Knoxville, Tennessee, USA

**Keywords:** Bartonella, cetacean, porpoise, emerging, stranded, research

## Abstract

We report detection of *Bartonella henselae* DNA in blood samples from 2 harbor porpoises (*Phocoena phocoena*). By using real-time polymerase chain reaction, we directly amplified *Bartonella* species DNA from blood of a harbor porpoise stranded along the northern North Carolina coast and from a preenrichment blood culture from a second harbor porpoise. The second porpoise was captured out of habitat (in a low-salinity canal along the northern North Carolina coast) and relocated back into the ocean. Subsequently, DNA was amplified by conventional polymerase chain reaction for DNA sequencing. The 16S–23S intergenic transcribed spacer region obtained from each porpoise was 99.8% similar to that of *B. henselae* strain San Antonio 2 (SA2), whereas both heme-binding phage-associated *pap*31 gene sequences were 100% homologous to that of *B. henselae* SA2. Currently, the geographic distribution, mode of transmission, reservoir potential, and pathogenicity of bloodborne *Bartonella* species in porpoises have not been determined.

## Abstract 2

DNA in porpoises suggests an emerging infectious disease in marine mammals.

Bartonellosis is a newly emerging worldwide zoonotic disease (*1**,**2*) that can be caused by a spectrum of *Bartonella* species. These members of the alpha subdivision of the class Proteobacteria are gram-negative aerobic bacilli and comprise at least 20 species and subspecies. Infection with *Bartonella* species causes lymphadenopathy (*3*), disorders of the central nervous system (including encephalopathy, hemiplegia, epilepsy, and subcortical frontoparietal lesions) (*4**–**7*), bacillary angiomatosis and bacillary peliosis (*8*), fever, adenitis, endocarditis (*9**–**12*), hepatosplenic involvement, cutaneous vasculitis, and osteomyelitis in domestic animals and humans (*13**–**15*).

*Bartonella* species have been isolated from numerous domestic and wild terrestrial animals, including cats, dogs, deer, cattle, lions, rabbits, and rodents (*16**–**20*). Because *Bartonella* spp. frequently induce persistent intravascular infections, particularly in reservoir hosts, attributing disease causation to *Bartonella* infection in animals or in human patients has been difficult, and satisfying Koch postulates for disease causation remains challenging (*21*).

Because conventional microbiologic techniques lack sensitivity, bartonellosis is usually diagnosed by using polymerase chain reaction amplification of organism-specific DNA sequences or serologic testing (*1**,**22**–**25*). Recently, a more sensitive isolation approach was developed by using *Bartonella* alpha proteobacteria growth medium (BAPGM) followed by real-time polymerase chain reaction (PCR). This method has greatly facilitated the molecular detection or isolation of *Bartonella* species from the blood of sick and healthy animals (*24*). The relative sensitivity of diagnostic methods used to detect *Bartonella* species infection greatly influences the ability to establish disease causation. The use of these optimized microbiologic techniques facilitated the recognition of bloodborne *Bartonella* spp. infections in porpoises, as reported in this study.

We report real-time PCR detection of *B. henselae* SA2 DNA in porpoise blood samples. Harbor porpoises are cetaceans in the family *Phocoenidae* that are found alone or in small groups in coastal waters, bays, and estuaries in cold, temperate, and subarctic waters of the Northern Hemisphere (*26*). In the western Atlantic, they range from Baffin Island and Labrador in the north, extending as far south as North Carolina in the winter. Initially, blood samples were screened by using real-time PCR targeting the *Bartonella* 16S–23S RNA intergenic spacer (ITS) region, or the heme-binding phage-associated gene *pap*31 (*27*). Preenrichment liquid BAPGM blood cultures were also screened by real-time PCR, after which conventional PCR was used to obtain DNA for sequencing and identifying *Bartonella* species to the strain level.

## Materials and Methods

### Sample Testing

Blood samples (anticoagulated with EDTA) were obtained from a live, stranded yearling female harbor porpoise (*Phocoena phocoena*) that required humane killing in northern North Carolina in May 2005 (MLC 001) and from a yearling male harbor porpoise. The male porpoise was captured out of habitat (in a low-salinity canal [3 parts salt per 1,000; by comparison, sea water contains 36 parts per 1,000] along the northern North Carolina coast) and relocated to the ocean in March 2005 (AAH 009). The samples were analyzed as follows. Following DNA extraction, real-time PCR (using DNA probes) and conventional PCR were used to screen for *Bartonella* DNA in each blood sample (*23*). A preenrichment culture was established from the original sample by using liquid BAPGM (*24*). After a 7-day incubation period, a sample was removed from the culture flask for conventional PCR and real-time PCR. In addition, sheep blood (used as blood supplement for preenrichment culture) and a sheep blood preenrichment BAPGM culture were screened for *Bartonella* DNA as a negative control at the time each porpoise sample was processed. *Bartonella* DNA was not detected in any control samples.

### Growth Medium

Preenrichment culture of EDTA-anticoagulated blood samples from the 2 porpoises tested in this study was performed as follows. A 1-mL aliquot of blood was added to 10 mL of BAPGM and incubated at 35°C in a 5% CO_2_, water-saturated atmosphere as previously described (*24*).

### DNA Extraction and PCR Screening of Blood and Blood Cultures

Screening for *Bartonella* species DNA was conducted by using conventional PCR and real-time PCR directly with EDTA-anticoagulated blood and from the 7-day postenrichment BAPGM blood culture samples. Gene sequencing was used to establish the species and strain classification. DNA was prepared by using the DNA Mini Kit (Qiagen, Valencia, CA, USA) from 200 μL of the blood sample and from 200 μL of preenrichment blood culture in BAPGM medium. After extraction, DNA concentration and quality were measured by using an absorbance ratio at 260:280 nm.

### Real-Time PCR Analysis

Real-time PCR screening for *Bartonella* species was performed as described previously (*23*). Scorpion 321 fluorescent probe 5´-FAM-CCG CGT TTT TCA AAG CCC ACG CGG-QUE-HEG-AGA TGA TGA TCC CAA GCC TTC TGG-3´ and oligonucleotide 425as 5´-GGA TRA AYY RGW AAA CCT TYM YCG G-3´ were used as sense and antisense primers, respectively, for screening of the *Bartonella* genus-specific ITS region. Identification of *Bartonella* species was conducted by using real-time multiplex PCR with species-specific fluorescent probes (Taqman, Appled Biosystems, Foster City, CA, USA) in conjunction with oligonucleotides 321s 5´-AGA TGA TGA TCC CAA GCC TTC TGG CG-3´ and 421as 5´-GGA TRA AYY RGW AAA CCT TYM YCG G-3´ as forward and reverse primers (Integrated DNA Technologies, Coralville, IA, USA), respectively. The species-specific fluorescent probe sequences (Taqman) used were 5´-FAM-CCA CCG TGG GCT TTG AAA AAC GCT-3´ DBHQ1 for *B. henselae*, 5´-TexRed-GGG ACT TTA AGG AAG ACA CTT TTG TG-BHQ2-3´ for *B. quintana*, 5´-ROX-TGC ACA AGC CTC TGA GAG GGA TGA ANG A-BHQ2-3´ for *B. clarridgeiae*, and Cy3 5´-CTT TCY TGT AAG AGT GTA TTT TTT ATC TAA GA-BHQ2-3´ for *B. vinsonii* subspecies *berkhoffii*. Real-time reactions were performed by using a SmartCycler II System (Cepheid, Sunnyvale, CA, USA) in 25-μL reaction volumes as described previously (*23*). Negative controls were prepared by using DNA extracted from sheep blood samples (the same used as blood supplement in BAPG liquid medium). PCR positive controls contained DNA from sheep blood samples spiked with *B. clarridgeiae* (ATCC 700095; American Type Culture Collection, Manassas, VA, USA), *Bartonella henselae* Houston-1 (ATCC 49882), *B. quintana* Fuller (ATCC VR-358), or *B. vinsonii* subsp. *berkhoffii* (ATCC 51672) DNA to a final concentration of 10 fg/μL. real-time PCR conditions were a single hot-start cycle at 95°C for 30 s, followed by 55 cycles of denaturing at 94°C for 10 s, annealing at 58°C for 6 s (for *Bartonella*) or at 60°C for 6 sec (for the 4 *Bartonella* species), and final extension at 72°C for 10 s. Positive amplicons were detected by reading fluorescence at the appropriate wavelength.

### Conventional PCR Analysis

#### ITS Region

PCR screening of the *Bartonella* 16S–23S ITS region was performed on samples that were positive by real-time PCR, as described (24), when amplicon size allows preliminary species identification. Oligonucleotides 321s 5´-AGA TGA TGA TCC CAA GCC TTC TGG-3´ and 983as 5´-TGT TCT YAC AAC AAT GAT GAT G-3´ were used as forward and reverse primers, respectively. The ITS region was amplified in a 25-μL reaction volume that contained 8.5 μL of molecular grade water (Epicentre, Madison, WI, USA) 0.5 μL 10 mmol/L dNTP mixture, 2.5 μL of 10× PCR buffer, 2.5 μL of 25 mmol/L MgCl_2_, and 0.7 units of Amplitaq Gold DNA polymerase (Applied Biosystems). The reaction mixture was completed by adding 0.25 μL of 30 μmol/L of each forward and reverse primer (Integrated DNA Technologies) and 10 μL of DNA from each sample tested. PCR negative controls contained 10 μL of DNA extracted from BAPGM (when testing BAPGM cultures) or 10 μL of DNA extracted from sheep blood (when testing blood samples). Conventional PCR conditions were a single hot-start cycle at 95°C for 5 min, followed by 45 cycles of denaturing at 94°C for 45 s, annealing at 54°C for 45 s, and extension at 72°C for 45 s. Amplification was completed by an additional cycle at 72°C for 5 min. Products were analyzed by electrophoresis on 2% agarose gels and detected by staining with ethidium bromide and viewing under UV light. Amplicon products were sequenced to identify species strains.

#### *pap*31 Gene Amplification

PCR species screening was performed by using primers designed to amplify a consensus sequence of the phage-associated gene *pap*31 found in several species of the *Bartonella* genus (*27*). Oligonucleotides Pap31 1(s) 5´-GAC TTC TGT TAT CGC TTT GAT TT-3´ and Pap31 688 (as) 5´-CAC CAC CAG CAA MAT AAG GCA T-3´ were used as forward and reverse primers, respectively. Amplification of the *pap*31 gene was performed in a 25-μL final volume reaction as described above. PCR conditions for *pap*31 gene amplification were a single hot-start cycle at 95°C for 5 min, followed by 45 cycles of denaturing at 94°C for 45 s, annealing at 58°C for 45 s, and extension at 72°C for 45 s. Amplification was completed by an additional cycle at 72°C for 5 min. Products were analyzed by electrophoresis on 2% agarose gels and detected by staining with ethidium bromide and viewing under UV light. Amplicons were sequenced to identify specific strains.

### Cloning and Sequencing of ITS and *pap*31 Gene Amplicons

*Bartonella* ITS and *pap*31 DNA sequencing was performed for 2 positive amplicons for both the ITS and *pap*31 gene to confirm the *Bartonella* species and strain. The amplified PCR products were cloned into the Plasmid pGEM-T Easy Vector System (Promega, Madison, WI, USA). Recombinants (white colonies) were selected on the basis of the right size of the insert in the plasmid using a plasmid miniprep procedure (Qiagen). Sequencing of plasmid inserts was done by Davis Sequencing, Inc. (Davis, CA, USA). Sequence analysis and alignment with GenBank sequences were performed by using AlignX software (Vector NTI Suite 6.0, InforMax, Inc., Frederick, MD, USA) to identify bacteria at species and strain level.

## Results

### Blood and Preenrichment Blood Culture Real-Time PCR

Following direct extraction from blood, *Bartonella* DNA was amplified from 1 (MLC 001) of the 2 porpoises. Real-time PCR amplification of *Bartonella* DNA was possible from the harbor porpoise stranded along the North Carolina coast, but not from the porpoise (AAH 009) that was captured and released until after the 7-day BAPGM enrichment period. Using a multiplex real-time PCR assay developed in the Vector-Borne Disease Diagnostic Laboratory at North Carolina State University, we identified *B. henselae* as the infecting species in both porpoises. *Bartonella vinsonii* subsp. *berkhoffii*, *B. clarridgeae*, and *B. quintana* DNA was not detected by using real-time PCR species-specific probes, which suggests that these species were not present in the original blood samples or in the preenrichment cultures. *Bartonella* DNA was not detected by real-time PCR in samples extracted from the sheep blood used as a negative control before and after preenrichment culture.

### Cloning and Sequencing

The *Bartonella* species was confirmed by cloning and sequencing the 16S–23S ITS region and the *pap*31 gene. The ITS and *pap*31 DNA sequences from both porpoises were consistent with that of *B. henselae* strain San Antonio 2 (GenBank accession no. AF369529). The 16S–23S ITS sequence matches were 675/677 bp (99.7%) for MLC 001 and 676/677 bp (99.8%) for AAH 009. The ITS sequence from MLC 001 contained 2 bp insertions (a C and a T), at positions 45 and 675, respectively, when compared with the ITS GenBank sequences for *B. henselae* SA2. Sequences from AAH 009 contained only a C insertion at position 45. The *pap*31 gene sequences derived from both porpoises matched 540/540 bp (100%) with *B. henselae* SA2 (GenBank accession no. AF308168).

## Discussion

We report the first detection of a *Bartonella* species from the blood of a cetacean, the detection of *B. henselae* from a nonterrestrial animal. This study was initiated by a request to use molecular diagnostic to evaluate blood from a stranded sick porpoise and from a porpoise captured out of its natural habitat.

MLC 001, a yearling female that was stranded in a remote location, may have been stranded for many hours before it could safely be reached and humanely killed. There was no evidence of fisheries interaction. The animal was thin, and necropsy showed no food in its stomach. Few obvious gross lesions and parasites were noted. The degree of postmortem autolysis was more than would be expected in a freshly killed animal. This finding, with signs of cranial epaxial and caudal abdominal muscle hemorrhages and the friable condition of the muscle, suggests that the animal's condition likely deteriorated during stranding. Results of histopathologic examination were unremarkable and lesions known to be consistent with bartonellosis were not found.

AAH 009, found swimming in a low-salinity canal, had 2 lacerations (2.5 and 5 cm long) over the left dorsal trunk cranial to the dorsal fin, 1 of which extended the full thickness through the blubber, but was not bleeding. In addition, ≈8 clusters of pinpoint lesions were observed slightly cranial and dorsal to the lacerations, which may have been due to punctures or viral skin lesions. Assessment of the porpoise's behavior, body condition, and hematologic parameters indicated that it was not debilitated despite its unusual location. It was then tagged and released into the ocean. Molecular evidence of *Bartonella* infection was obtained by direct PCR on whole blood from MLC 001, and by using a recently improved diagnostic method that combines preenrichment blood culture (*24*) and real-time PCR in samples from AAH 009. An isolate was not obtained from either porpoise after application of the preenrichment BAPGM blood culture onto a blood agar plate.

Recently, various emerging or reemerging infectious diseases or infections of serious epizootic or zoonotic potential have been described in marine mammals (*28**–**32*). These include morbillivirus infection, brucellosis, toxoplasmosis, sarcocystosis, papillomavirus infection, and West Nile virus infection (*29**,**31**,**33*). As was the case for brucellosis, recognized as an emerging disease in a marine mammal in 1994 (*34**,**35*), bartonellosis may become an important emerging marine mammal infectious disease.

Current evidence indicates that all known *Bartonella* species are vector transmitted, with bites or scratches (cat scratch disease) providing an alternative means of transmission for *B. henselae* (*2**,**20*). The *B. henselae* SA2 sequence deposited in GenBank was amplified from an isolate derived from a human lymph node aspiration sample (*9*). Although exposed to rose thorns and cats, the patient did not recall an animal bite or scratch, and illness developed after a recent tick bite, which was attributed to *Amblyomma americanum*, based on the location and time of the year. Since the mode of infection was not established in these porpoises, the potential role of trauma, such as induced by tooth raking, or transmission via biting marine invertebrates should be investigated.

As for many fastidious pathogens, difficulties associated with *Bartonella* detection and isolation have compromised efforts to define the role of these organisms in disease causation. Enhancement of organism-specific DNA detection and isolation through the use of an optimized isolation medium such as BAPGM can aid in evaluating serodiagnostic assays and may advance understanding the diversity, adaptation, and epidemiology of this genus (*24**,**36*). Based on the recent use of BAPGM in the North Carolina State University Vector-Borne Disease Diagnostic Laboratory, we believe that chronic infection with *Bartonella* species can contribute to subtle clinical abnormalities or vague symptoms in companion and wild animals or in humans.

Angiomatosis, an important pathologic manifestation of *Bartonella* infection in immunocompromised persons (*8**,**21*), has been previously reported in bottlenose dolphins (*37*). Since angiomatous lesions are unusual pathologic lesions and *B. henselae* is a recently recognized cause of vasoproliferative lesions in humans (*8**,**15*), examination of angiomatous lesions from cetaceans for the presence of *B. henselae* should be a priority in future studies. In addition, the involvement of *Bartonella* species in disorders of the central nervous system and neurologic dysfunction in animals and humans (*4**–**7*) suggests that this genus should be considered in stranding events. Although *Bartonella* infection in the vasculature of reservoir hosts is generally not accompanied by pathologic changes, *Bartonella* spp. may become pathogenic in combination with severe stress, malnutrition, increased exposure to toxins, and concurrent infection with other organisms. PCR amplification directly from an extracted blood sample or from a preenrichment blood culture showed that both porpoises in this study were infected with *B. henselae* SA2. Since infection with *Bartonella* spp. has been documented in a marine mammal, the clinical impact, mode of transmission, pathology, and epidemiology are areas for additional inquiry.
